# COVID-19 home remedy consumption and perceived effectiveness among adult population in Brunei Darussalam: a PLS-SEM approach

**DOI:** 10.1186/s12906-024-04374-9

**Published:** 2024-02-12

**Authors:** Siti Nurzaimah Nazhirah Zaim, Deeni Rudita Idris, Hanif Abdul Rahman

**Affiliations:** PAPRSB Institute of Health Sciences, Universiti Brunei Darussalam, Tungku Link Road, Gadong, BE1410 Brunei Darussalam

**Keywords:** COVID-19, Home remedy, Complementary and alternative medicine, Prevalence, Nursing, PLS-SEM

## Abstract

**Background:**

The Coronavirus Disease 2019 (COVID-19) caused by SARS-CoV-2 affects the respiratory system and causes severe acute respiratory infections. Several cultures have influenced the use of home remedies to ease mild symptoms of COVID-19 sometimes alongside conventional medicine. The aim of this study was to investigate the usage of COVID-19 home remedies among the adult population in Brunei.

**Methods:**

The study design was a cross-sectional study using an online survey and distributed nationwide. The statistical analysis of the data included descriptive statistics describing the sociodemographic characteristics of the respondents, their experience with COVID-19 infection, consumption of general home remedies and COVID-19 home remedy, their practice of COVID-19 home remedy use, and their attitude towards the general use of home remedy. Sub-group analysis including Pearson's chi-square test and Fisher's exact test was computed for the variables in relation to the COVID-19 home remedy use and the perceived effectiveness of the types of home remedy. Multivariate analysis including Partial Least Squares Structural Equation Modelling (PLS-SEM) was applied to determine the correlations between the factors and outcomes measured. *P*-value less than 0.05 is considered statistically significant.

**Results:**

There was a total of 864 eligible responses included in the study. The primary findings showed COVID-19 home remedy was used by 72.2% of the study participants. Most frequently used types of COVID-19 home remedy were cloves (37%), lemon (37%), and honey (23%). There was an infrequent usage of coconut (4.6%), eucalyptus oil (3.7%), *habbatussauda* (3.5%), traditional/ herbal cough syrup (2.4%), and turmeric (2.2%). The PLS-SEM demonstrated that ‘Experience’ appears to be a central endogenous variable that affects the decision on the use of COVID-19 home remedy. This relationship is mediated by ‘Safety’, ‘Source’, and ‘Opinion’ which have significant contributions to the outcome, accounting for 98.2% of the variance explained (R-square = 0.982).

**Conclusions:**

The outcome of this study establishes the baseline prevalence of COVID-19 home remedy consumption among Bruneian residents and compared the previous study on Brunei’s general use of traditional medicine. The present findings could help nurses and other healthcare professionals in Brunei understand the practice of Bruneian adults on the consumption of home remedies for COVID-19.

## Introduction

### Background

Traditionally passed down across generations, home remedies, classified as Complementary and Alternative Medicine (CAM), address minor health issues. The origins of these remedies vary among cultures. The SARS-CoV-2 virus, causing COVID-19, primarily affects the respiratory system. With widespread COVID-19 vaccination, most cases now exhibit mild upper respiratory symptoms. Various cultures and religions embrace home remedies and complementary therapies to alleviate mild COVID-19 symptoms, sometimes adjunctive to conventional medicine. Nurses, as promoters of holistic care, play a role in transcultural nursing, understanding patients’ cultural preferences for remedies in managing COVID-19 guiding evidence-based self-management education.

### COVID-19 Overview

In late 2019, a novel coronavirus disease emerged in Wuhan City, Hubei Province, China [1]. The World Health Organization officially designated it a global pandemic on March 11, 2020, after careful consideration [2]. As of July 26, 2023, the cumulative global tally stood at 768,560,727 confirmed cases with 6,952,522 reported deaths [3]. Brunei Darussalam recorded its first 2019-nCoV case in March 2020, responding with a Movement Control Order to curb the spread of virus. Despite an apparent halt in community transmission in May 2020, new COVID-19 cases emerged in early August 2021, signalling the onset of the second wave and leading to a partial lockdown [4, 5]. As of July 26, 2023, Brunei reported 309,561 confirmed COVID-19 cases with 2 deaths [6]. Approaching its fourth year, COVID-19 is no longer classified as global public health emergency but acknowledged as an enduring health challenge.

### Home remedy definition

Defining “home remedies” poses a challenge due to cultural variations, with terms like traditional medicine, fold medicine, CAM, and natural remedies used interchangeably. Cultural and social factors influence the utilisation of home remedies, which may be viewed as self-management. This study considers home remedies, incorporating identified terms and specifying ingredients found at home for treating illnesses.

### Role of nurses

In nursing, acknowledging patients’ cultural practices and traditional medicine is advantageous. Despite debates on traditional medicine’s scientific validity, overlooking patients’ cultural context may diminish care quality [7]. Nurses should consider patients’ use of home remedies, but must also inform them about the risk, benefits and contradictions with conventional medicine. Respecting patient autonomy involves promoting choices in treatment plans, but not disclosing risks may lead to harm [8]. In South Africa, discussing herb-drug interaction with patients is challenging due to nurses’ limited herbal medicine knowledge or patients’ non-disclosure on their herbal medicine consumptions [9]. Amid resource shortages in the pandemic, understanding local home remedies becomes crucial for nurses to ensure safe practices.

### Existing literature and research gap

Globally, numerous experiments explored natural sources for potential COVID-19 remedies. In Brunei, studies on home remedies exists in the form of ethnomedicine, yet none specifically address COVID-19. A cross-sectional study examines the prevalence of general traditional medicine use [10], while another investigates prevalence and predictive factors for CAM use [11].

### Aim & objectives

The aim was to investigate the usage of COVID-19 home remedies among the adult population in Brunei. The objectives were: 1) To estimate the prevalence of home remedy usage for COVID-19 among Brunei’s adult population; 2) To establish the perception, attitude, and practice of utilising home remedy for COVID-19 among Brunei’s adult population in relation to sociodemographic factors; 3) To investigate, by demographic stratification, the prevalence as well as the perception, attitude, and practice of COVID-19 home remedies; and 4) To develop a structural model based on the perception, attitude, and practice data to explain home remedy usage in the adult Brunei population.

### Implications of research

This research seeks to bridge the gap between traditional and modern medicine, offering valuable insights into home remedies used by Bruneian residents and its efficacy for COVID-19. The study’s outcomes will the Ministry of Health and stakeholders, guiding health education for those favouring home remedies. Additionally, it will assist nurse educators and professionals in formulating guidelines for COVID-19 patient care, enhancing understanding of prevalent home remedies and Bruneian adults’ attitudes and beliefs. The findings will also contribute to researchers’ comprehension of Bruneian adults’ health-seeking behaviour and treatment intentions.

Significant findings derived from this study have the potential to contribute to: the formulation of evidence-based guidelines for healthcare professionals regarding the utilisation of COVID-19 home remedies; fostering comprehension of societal cultures and lifestyles, thereby cultivating acceptance of the utilisation of COVID-19 home remedies and traditional medicine in conjunction with conventional medical practices among healthcare personnel; and developing a comprehensive citizen’s guide aimed at assisting individuals afflicted by COVID-19 in the proper and secure self-care practices.

### Literature review

A literature review explored existing evidence on COVID-19 patients using home remedies for manging mild symptoms. Employing a PEO format, review focused on literature published in English between 2018 to 2022. Searches were conducted on PubMed Central, Springer Link, and Science Direct to identify research gaps.

### Summary of literature review

An literature overview identified two main themes regarding home remedy use for managing COVID-19 symptoms: studies on traditional medicinal plants, and the utilisation of CAM.

### Studies on traditional medicinal plants

Literature findings suggest an initial exploration of numerous plants traditionally used, now under review and experimentation for potential medicinal properties in the treatment of COVID-19. African traditional medicine, encompassing Cameroonian and West African medicinal plants, was among the recognised established traditional remedies mentioned [12–14]. One review centred on a Saudi Arabian medicinal plant, commonly known as Black Seed [15], another explored traditional medicine in Iran [16], and several others examined essential oil in Brazil [17] and India [18]. Studies conducted in India explored the potential properties of natural products [19], traditional medicinal plants [20], the efficacy of Siddha Medicinal decoctions [21], and Ayurveda Rasayana for antiviral effects and immune system enhancement [22]. Lastly, an investigation on the efficacy and mechanism of a Chinese herb [23]. This indicates that numerous literary works have already explored the study of traditional medicine derived from plants.

### Use of complementary and alternative medicine

Mentioned CAM modalities include Ayurvedic Medicine, Chinese Traditional Medicine, and Siddha Medicine. A study addressed CAM’s role in preventing and treating COVID-19 [24], while others explored CAM use alongside allopathic drugs [25–27], and natural pharmacy’s role in relief and prevention [24, 28–30]. One study examined CAM prevalence and predictive factors during the first COVID-19wave [31]. Overall, the literature highlights the CAM utilisation not only for symptoms management but also perceived prevention of COVID-19.

## Methods

### Study design, population and sample

The study employed a cross-sectional design via an online survey with an estimated sample size based on Brunei’s total recovered COVID-19 cases (160,954 cases as of July 1, 2022). The required sample size was considering ± 5% margin of error, 50% prevalence, 95% confidence interval, and a 20% potential loss. I anticipated 95% CI is (45%, 55%),calculated using Scalex SP calculator [32]. The eligibility criteria for participants were as follows.

### Inclusion criteria

The target participants were: 1) People who were infected with COVID-19 in Brunei Darussalam between March 2020 to February 2023; 2) People who used home remedies with or without the use of modern medicine during COVID-19 infection; 3) Aged 18 and above; and 4) Citizens and permanent residents of Brunei Darussalam.

### Exclusion criteria

Meanwhile, the exclusion criteria was other respiratory infections.

### Data collection, tools, and procedure

#### Data collection process

Pretesting for questionnaire comprehensibility occurred from December 6 to December 12, 2022, involving 10 purposely selected participants, from science faculties (i.e., PAPRSB Institute of Health Sciences, Faculty of Science, and Faculty of Integrated Technology) and non-science faculties (i.e., Faculty of Arts and Social Sciences, and School of Business and Economics) at the University among the students and staff. The survey underwent amendments, addressing spelling errors and missing translations before the main data collection, conducted online from January 16 to February 26, 2023, on Qualtrics, a platform for online questionnaire, to minimised COVID-19 risk and maintain participants’ anonymity.

### Data collection tool

Data collection utilised a self-developed survey drawing from literature and a cross-sectional survey on patients’ general home remedy use in Germany [33] and a Brunei Darussalam study on traditional medicine prevalence [10]. Permission to adapt to the Brunei study was obtained. The questionnaire covers three sections: socio-demographic, participants’ COVID-19 experiences, and home remedy consumption during COVID-19 infections. To ensure participant eligibility, the online questionnaire was designed to only accept the responses of those who are eligible for the survey using the ‘Display Logic’ and ‘Skip Logic’ functions available on the platform.

### Main recruitment process

After ethical approval from the university, and permission from the four Brunei District Office (Brunei-Muara, Tutong, Belait, and Temburong), the survey link was disseminated nationwide. Gatekeepers from the District Offices received emails explaining the dissemination process. The gatekeepers then digitally shared a poster containing the survey link and research details with the *Penghulu* (Chiefs), who then circulated it to all Village Heads in their respective *Mukims* (subdivisions of a district), reaching members of the village.

### Data analysis

Data collected from the online questionnaire was extracted from Qualtrics and imported into Microsoft Excel for the process of data analysis. The statistical analysis of the data included descriptive statistics used to describe the sociodemographic characteristics of the respondents (age, gender, race, education level, employment status, monthly income, religion, and marital status), their experience with COVID-19 infection (symptoms experienced and hospitalisation), consumption of general home remedy and COVID-19 home remedy, their practice of COVID-19 home remedy use (with modern medicine, vitamins or supplements, source of ingredients, reason, knowledge, types of home remedy, and perceived effectiveness), and their attitude towards the general use of home remedy (perceived safety, reason for perceived safety, and opinion on home remedy). Sub-group analysis including Pearson's chi-square test and Fisher's exact test was computed for the variables in relation to the COVID-19 home remedy use and the perceived effectiveness of the types of home remedy. Multivariate analysis including Partial Least Squares Structural Equation Modelling (PLS-SEM) was applied to determine the correlations between the factors ‘Experience’, ‘Opinion’, ‘Safety’, ‘Source’, ‘Reason’ and ‘Knowledge’, and outcomes measured, ‘Home Remedy Use’ and ‘Effective’. Attitude and opinion refers to their perceived effectiveness of the types of home remedy they are using, the perceived safety of home remedy and their opinion of how effective the COVID-19 home remedy would be with modern medicine. Practice refers to their previous experience of using home remedy in general, where they get their supply of home remedy ingredients and their reasons for using the home remedy. All statistical analysis was computed using R version 4.2.1 (2022–06-23) and RStudio 2022.07.1 + 554. P-value less than 0.05 is considered statistically significant.

The selection of Partial Least Squares Structural Equation Modelling (PLS-SEM) for the data analysis was motivated by the unique capability of this method to yield results that were unattainable through alternative methodologies. The study comprised of two distinct outcomes and related factors. Therefore, further to univariate analysis that explores factors to each outcome, structural equation modelling (SEM) was used to establish multivariate link between all the factors to both outcomes, simultaneously. Upon further exploration, PLS-SEM was suitable with the structure and distribution of data of this study.

### Ethical consideration

Ethics approval was obtained from the Ethics Committee of PAPRSB Institute of Health Sciences (UBD/PAPRSBIHSREC/2022/31) on December 6, 2022. Permission to extract data was obtained from the four District Offices in Brunei Darussalam. Approval to recruit pretesting samples was obtained from the Director, Office of Vice-Chancellor (Research)/ Chair, University Research Ethics Committee (UREC) of Universiti Brunei Darussalam. All participant-identifying information such as name and any identification number, was not collected to ensure participants’ anonymity and confidentiality. Online data collection ensured no direct contact with research participants. Informed consent was obtained before starting the online questionnaire, and participants need to click the “I agree” statement or “I decline” if they refuse to participate. The dataset stored on a password-protected computer was only accessible by the research team and will be deleted five years after the completion of study.

## Results

Table 1 outlines the participant characteristics of the participants with 864 eligible responses. The majority, 45%, falls into the 25 to 34 age group. Gender distribution was 59% female and 41% male, while 86% were Malay. Educational levels comprise 79% higher education, and 21% secondary school and sixth form. Private sector employees or self-employed constitute 40%, government servants represents 36%, and the rest were unemployed and others (24%). Monthly income distribution: More than B$2000 (50%), B$500 to B$2000 (26%), and less than B$500 (24%). Almost all respondents practice Islam (98%), with 57% single and 43% married.

**Table 1:** Sociodemographic characteristics of respondents (*N* = 864)

Characteristics	N (%)
**Age**	
18 – 24	139 (16%)
25 – 34	391 (45%)
35 – 44	158 (18%)
45 +	176 (20%)
**Gender**	
Male	350 (41%)
Female	514 (59%)
**Race**	
Malay	747 (86%)
Others	117 (14%)
**Education Level**	
Secondary School & Sixth Form	179 (21%)
Higher Education	685 (79%)
**Employment Status**	
Private Sector Employee or Self-employed	347 (40%)
Government Servant	310 (36%)
Unemployed & others	207 (24%)
**Monthly Income**	
< B$500	210 (24%)
B$500 – B$2000	225 (26%)
> B$2000	429 (50%)
**Religion**	
Islam	847 (98%)
Others	17 (2.0%)
**Marital Status**	
Single	496 (57%)
Married	368 (43%)

Table 2 displayed participants’ COVID-19 experience and disease severity. The majority, 94% reported symptomatic infection, with only 1.9% requiring hospitalisation. This suggests a prevalence of mild COVID-19 cases among the surveyed participants.

**Table 2:** Experience of COVID-19 infection amongst the respondents (*N* = 864)

COVID-19 Infection Experience	N (%)
**Symptomatic**	
Yes	808 (94%)
No	56 (6.5%)
**Hospitalisation**	
Yes	16 (1.9%)
No	848 (98%)

Table 3 reveals comparable prevalence between COVID-19 home remedy and general home remedy consumption. 70% of respondents reported prior general home remedy use, while 72.2% indicated using home remedies for COVID-19.

**Table 3:** Prevalence of COVID-19 home remedy use and general home remedy use (*N* = 864)

Home Remedy Consumption	N (%)
**For General Use**	
Yes	602 (70%)
No	262 (30%)
**For COVID-19 Use**	
Yes	624 (72.2%)
No	240 (27.8%)

Table 4 reported a higher prevalence of participants using COVID-19 home remedies alongside modern medicine (82%) and supplements/vitamins (78%) compared to exclusive use. This implies the primary role of COVID-19 home remedies as complementary and integrative therapy. The ingredients were mainly sourced from the supermarkets (40%) and given by friends/family (26%) with minimal contributions from personal garden (3.5%), Chinese dispensary (2.4%), overseas purchases (1.1%), or traditional/spiritual healers (1.1%). Motivations were intrinsic, focused on symptoms relief (41%) rather than as a perceived cure to the infection (19%) or to prevent a more severe infection (19%). Externally, family recommendations (32%) influenced usage more than non-family recommendations (16%). Knowledge sources included passed down from previous generations (36%), social media (30%), non-family recommendations (29%), internet searches (24%), self-learning (20%), and less from books/ magazine (3.4%). Commonly used COVID-19 home remedies included cloves (37%), lemon (37%), and honey (23%), with infrequent use of coconut (4.6%), eucalyptus oil (3.7%), *habbatussauda* (3.5%), traditional/ herbal cough syrup (2.4%), and turmeric (2.2%). Among users of COVID-19 home remedy, only 11% perceived the home remedies used as effective or very effective.

**Table 4:** Respondents’ practice of COVID-19 home remedy use (*N* = 624)

Practice of COVID-19 home remedy use	N (%)
**Taken together with modern medicine?**	
Yes	513 (82%)
No	111 (18%)
**Taken together with vitamins or supplements?**	
Yes	484 (78%)
No	140 (22%)
**Ingredient Source**	
Supermarket	247 (40%)
Given by friends/family	160 (26%)
My garden pot	22 (3.5%)
Chinese Dispensary	15 (2.4%)
Buy from overseas	7 (1.1%)
Traditional/ Spiritual Healers	7 (1.1%)
**Reason for using COVID-19 home remedy:** I used home remedies …	
… to relief/ ease the symptoms experienced	256 (41%)
… as recommended by my family	197 (32%)
… as a cure for the infection	117 (19%)
… to prevent a more severe infection	116 (19%)
… as recommended by other people	102 (16%)
**Knowledge of COVID-19 home remedy:** My source of knowledge for the home remedies came from …	
… family members (including passed down from generation to generation)	233 (36%)
… social media	194 (30%)
… recommendations from someone I know (not family members)	184 (29%)
… internet searches	154 (24%)
… self-learning	126 (20%)
… books/ magazine	22 (3.4%)
**Type of COVID-19 home remedy:**	
Clove	231 (37%)
Lemon	231 (37%)
Honey	144 (23%)
Coconut	29 (4.6%)
Eucalyptus oil	23 (3.7%)
*Habbatussauda*	22 (3.5%)
Traditional/Herbal Cough Syrup	15 (2.4%)
Turmeric	14 (2.2%)
**Perceived Effectiveness**	
Not effective or less effective	771 (89%)
Effective or very effective	93 (11%)

Table 5 indicates varying perceptions of home remedy safety among participants. While 48% find general home remedy usage safe, others are uncertain or consider it unsafe. Among those perceiving safety (34%), the main reason is its natural source. Regarding effectiveness, 40% express uncertainty about home remedy compared to modern medicine, with some believing it works better with modern medicine (17%), just as well (16%), or less effectively (9.5%). Only 2.9% believes home remedies are more effective alone.

**Table 5:** Attitude of general home remedy use (*N* = 864)

Attitude of home remedy use	N (%)
**Perceived Safety:**	
Yes	288 (48%)
No	152 (25%)
Not Sure	162 (27%)
**Reasons of Perceived Safety:**	
Contains natural sources	204 (34%)
Has been practiced traditionally over the decades	14 (2.3%)
**Opinion on home remedy:**	
Do not know	345 (40%)
Home remedies work better with modern medicines	148 (17%)
Home remedies work just as well as modern medicine	139 (16%)
Modern medicines works better than home remedies	82 (9.5%)
Home remedies work better alone	25 (2.9%)
Others	125 (14%)

### Subgroup comparison between COVID-19 home remedy use and perceived effectiveness

Table 6 showed significant associations with COVID-19 home remedy use in gender (*p* < 0.001), race (*p* = 0.001), education level (*p* < 0.001), employment status (*p* < 0.001), monthly income (*p* < 0.001), religion (*p* < 0.001), and marital status (*p* = 0.001). No significant associations were observed regarding the participants’ sociodemographic characteristics and the perceived effectiveness of COVID-19 home remedy. Among non-users of COVID-19 home remedy (*N* = 240), 80% were female, 92% were Malay, 87% had higher education, and 41% were government servants. Most non-users had a monthly income of over B$2000 (43%), practiced Islam (93%), and married (51%). Of users (*N* = 624), 52% were female, 84% are Malay, 76% had higher education, and 45% were from the private sector or self-employed. Higher income correlates with increased COVID-19 home remedy use. All users practiced Islam, and 61% were unmarried.

**Table 6:** Respondents’ sociodemographic characteristics in relation to COVID-19 home remedy use and perceived effectiveness

	**COVID-19 Home Remedy Use (*N* = 864)**		***p*-value**	**Perceived Effectiveness (*N* = 624)**		***p*-value**
**Variables**	**No** (*N* = 240)	**Yes** (*N* = 624)		**Not or Less Effective** (*N* = 531)	**Effective or Very Effective** (*N* = 93)	
**Age**			0.113^a^			0.973 ^a^
18 – 24	43 (18%)	96 (15%)		82 (15%)	14 (15%)	
25 – 34	111 (46%)	280 (45%)		240 (45%)	40 (43%)	
35 – 44	32 (13%)	126 (20%)		106 (20%)	20 (22%)	
45 +	54 (22%)	122 (20%)		103 (19%)	19 (20%)	
**Gender**			** < 0.001**^a^			0.998 ^*a*^
Male	48 (20%)	302 (48%)		257 (48%)	45 (48%)	
Female	192 (80%)	322 (52%)		274 (52%)	48 (52%)	
**Race**			**0.001**^a^			0.589 ^a^
Malay	222 (92%)	525 (84%)		445 (84%)	80 (86%)	
Others	18 (7.5%)	99 (16%)		86 (16%)	13 (14%)	
**Education Level**			** < 0.001**^a^			0.107 ^a^
Secondary School and Sixth Form	32 (13%)	147 (24%)		119 (22%)	28 (30%)	
Higher Education	208 (87%)	477 (76%)		412 (78%)	65 (70%)	
**Employment Status**			** < 0.001**^a^			0.523 ^*a*^
Private Sector Employee or Self-employed	69 (29%)	278 (45%)		233 (44%)	45 (48%)	
Government Servant	98 (41%)	212 (34%)		180 (34%)	32 (34%)	
Unemployed & others	73 (30%)	134 (21%)		118 (22%)	16 (17%)	
**Monthly Income**			** < 0.001**^a^			0.410 ^a^
< B$500	94 (39%)	116 (19%)		95 (18%)	21 (23%)	
B$500 – B$2000	42 (18%)	183 (29%)		154 (29%)	29 (31%)	
> B$2000	104 (43%)	325 (52%)		282 (53%)	43 (46%)	
**Religion**			** < 0.001**^b^			
Islam	223 (93%)	624 (100%)		531 (100%)	93 (100%)	
Others	17 (7.1%)	0 (0%)				
**Marital Status**			**0.001**^a^			0.567 ^a^
Single	117 (49%)	379 (61%)		325 (61%)	54 (58%)	
Married	123 (51%)	245 (39%)		206 (39%)	39 (42%)	

Bold *p*-value = statistically significant at 0.05 level

^*a*^ Pearson’s Chi-squared test

^*b*^ Fisher’s exact test

There were no significant associations reported in Table 7 between COVID-19 home remedy consumption as well as the perceived effectiveness of COVID-19 home remedy with the severity of COVID-19 infection.

**Table 7:** Severity of COVID-19 infection in relation to the usage of COVID-19 home remedy and perceived effectiveness

	**COVID-19 Home Remedy Use** (*N* = 864)		***p*-value**	**Perceived Effectiveness (*N* = 524)**		***p*-value**
**Variables**	**No** (*N* = 240)	**Yes** (*N* = 624)		**Not or Less Effective** (*N* = 531)	**Effective or Very Effective** (*N* = 93)	
**Did you experience any symptoms while having the COVID-19 infection?**			0.891^a^			0.069 ^a^
Yes	224 (93%)	584 (94%)		493 (93%)	91 (98%)	
No	16 (6.7%)	40 (6.4%)		38 (7.2%)	2 (2.2%)	
**Have you ever been hospitalized due to COVID-19 infection?**			0.577 ^b^			0.114 ^b^
Yes	3 (1.3%)	13 (2.1%)		9 (1.7%)	4 (4.3%)	
No	237 (99%)	611 (98%)		522 (98%)	89 (96%)	

Bold *p*-value = statistically significant

^*a*^ Pearson’s Chi-squared test

^*b*^ Fisher’s exact test

Table 8, revealed significant associations between COVID-19 home remedy consumption and experience with general home remedy use (*p* < 0.001), safety perception due to traditional practice (*p* < 0.001), and home remedy opinion (*p* < 0.001). A notable association exists between perceived safety due to natural sources and the perceived effectiveness of COVID-19 home remedy (*p* = 0.044). Most COVID-19 home remedy users have prior general home remedy experience (93%), while non-users lack the experience of using general home remedy (92%). Almost half of the COVID-19 home remedy users perceived it as safe (49%), contrasting with all non-users of COVID-19 home remedy perceiving is as unsafe. Over half of the COVID-19 home remedy users were uncertain about home remedy efficacy compared to or in addition to modern medicine (54%), while non-users of COVID-19 home remedy think it works (61%). Those perceiving safety in natural sources finds COVID-19 home remedy ineffective or less effective (36%), while others find it effective or very effective (25%).

**Table 8:** COVID-19 home remedy use and perceived effectiveness in comparison to practice and attitude towards use of home remedy in general

	**COVID-19 Home Remedy Use**			**Perceived Effectiveness**		
**Variables**	**No** (*N* = 240)	**Yes** (*N* = 624)	***p*-value**	**Not / Less Effective** (*N* = 531)	**Effective / Very Effective** (*N* = 93)	***p*-value**
**Have you ever taken home remedies (in general) before?**			** < 0.001** ^a^			0.391 ^a^
Yes	19 (7.9%)	583 (93%)		498 (94%)	85 (91%)	
No	221 (92%)	41 (6.6%)		33 (6.2%)	8 (8.6%)	
**Do YOU feel that home remedies are safe?**			** < 0.001** ^a^			0.196 ^a^
Yes	0 (0%)	288 (49%)		239 (48%)	49 (58%)	
Not Sure	0 (0%)	162 (28%)		140 (28%)	22 (26%)	
No	19 (100%)	133 (23%)		119 (24%)	14 (16%)	
**Perceived Safety Reason:**						
Has been practiced traditionally over the decades	0 (0%)	8 (1.4%)	** < 0.001** ^b^	6 (1.2%)	2 (2.4%)	0.330 ^b^
Containing natural sources	0 (0%)	200 (34%)	0.230^a^	179 (36%)	21 (25%)	**0.044** ^a^
**Opinions on home remedy:**			** < 0.001** ^a^			0.204 ^b^
Home remedies work better with modern medicines	146 (61%)	2 (0.3%)		2 (0.4%)	0 (0%)	
Home remedies work better alone	22 (9.2%)	3 (0.5%)		2 (0.4%)	1 (1.1%)	
Home remedies work just as well as modern medicine	45 (19%)	94 (15%)		82 (15%)	12 (13%)	
Modern medicines works better than home remedies	0 (0%)	82 (13%)		63 (12%)	19 (20%)	
Do not know	5 (2.1%)	340 (54%)		291 (55%)	49 (53%)	
Others	22 (9.2%)	103 (17%)		91 (17%)	12 (13%)	

Bold *p*-value = statistically significant

^*a*^ Pearson’s Chi-squared test

^*b*^ Fisher’s exact test

Table 9 illustrates significant associations between the perceived effectiveness of COVID-19 home remedy and various ingredient sources, including those obtained from the supermarket (*p* < 0.001), given by friends/family (*p* < 0.001), personal gardens (*p* < 0.001), Chinese dispensary (*p* = 0.003), and purchases from overseas (*p* < 0.001). Furthermore, significant associations were observed with specific reasons for using COVID-19 home remedy, such as relieving symptoms (*p* < 0.001), family recommendations (*p* < 0.001), viewing it as cure (*p* < 0.001), and preventing severe infection (*p* < 0.001). All listed knowledge sources exhibit significant associations with the perceived effectiveness of COVID-19 home remedy. Additionally, certain types of COVID-19 home remedies, including clove (*p* < 0.001), lemon (*p* < 0.001), coconut (*p* < 0.001), eucalyptus oil (*p* < 0.001), traditional/ herbal cough syrup (*p* = 0.003), and turmeric (*p* = 0.002), show significant associations with perceived effectiveness.

**Table 9:** Practice of COVID-19 home remedy *(N* = 624)

Variables	Perceived Effectiveness (*N* = 624)		*p*-value
	**Not / Less Effective (*N* = 531)**	**Effective / Very Effective (*N* = 93)**	
**Did you take the home remedy together with modern medicine?**			0.298^a^
Yes	433 (82%)	80 (86%)	
No	98 (18%)	13 (14%)	
**Did you take the home remedy together with vitamins or supplements?**			0.298^a^
No	408 (77%)	76 (82%)	
Yes	123 (23%)	17 (18%)	
**Ingredient source**			
Supermarket	182 (34%)	65 (70%)	** < 0.001** ^a^
Given by friends/family	95 (18%)	65 (70%)	** < 0.001** ^a^
My garden pot	8 (1.5%)	14 (15%)	** < 0.001** ^b^
Chinese Dispensary	8 (1.5%)	7 (7.5%)	**0.003**^b^
Buy from overseas	0 (0%)	7 (7.5%)	** < 0.001** ^b^
Traditional/ Spiritual Healers	7 (1.3%)	0 (0%)	0.602 ^b^
**Reason for using COVID-19 home remedy:** I used home remedies …			
… to relief/ ease the symptoms experienced	170 (32%)	86 (92%)	** < 0.001** ^a^
… as recommended by my family	133 (25%)	64 (69%)	** < 0.001** ^a^
… as a cure for the infection	74 (14%)	43 (46%)	** < 0.001** ^a^
… to prevent a more severe infection	73 (14%)	43 (46%)	** < 0.001** ^a^
… as recommended by other people	81 (15%)	21 (23%)	0.078^a^
**Knowledge of COVID-19 home remedy:** My source of knowledge for the home remedies came from …			
… family members (including passed down from generation to generation)	148 (28%)	79 (85%)	** < 0.001** ^a^
… social media	129 (24%)	65 (70%)	** < 0.001** ^a^
… internet searches	103 (19%)	51 (55%)	** < 0.001** ^a^
… recommendations from someone I know (not family members)	131 (25%)	49 (53%)	** < 0.001** ^a^
… self-learning	82 (15%)	36 (39%)	** < 0.001** ^a^
… books/ magazine	7 (1.3%)	15 (16%)	** < 0.001** ^b^
**Type of COVID-19 home remedy:**			
Clove	167 (31%)	64 (69%)	** < 0.001** ^a^
Lemon	167 (31%)	64 (69%)	** < 0.001** ^a^
Coconut	0 (0%)	29 (31%)	** < 0.001** ^b^
Honey	116 (22%)	28 (30%)	0.081^a^
Eucalyptus oil	8 (1.5%)	15 (16%)	** < 0.001** ^b^
Traditional/ Herbal Cough Syrup	8 (1.5%)	7 (7.5%)	**0.003**^b^
Turmeric	7 (1.3%)	7 (7.5%)	**0.002**^b^
Habbatussauda	22 (4.1%)	0 (0%)	0.060 ^b^

Bold *p*-value = statistically significant

^*a*^ Pearson’s Chi-squared test

^*b*^ Fisher’s exact test

Among those perceiving COVID-19 home remedy as ineffective or less effective, ingredients were mostly sourced from the supermarket (34%) and given by friends or family (18%), primarily for symptom relief (32%) and family recommendations (25%), with knowledge on home remedy from family members (28%), other people (25%), social media (24%), internet searches (19%), self-learning, and books/magazines (1.3%). They mostly use cloves (31%) and lemon (31%).

In contrast, those perceiving COVID-19 home remedy as effective or very effective, mainly obtained ingredients from the supermarket (70%) and friends or family (70%), emphasising symptom relief (92%) and family recommendations (69%), with prevalent knowledge of home remedies from family members (85%), social media (70%), internet searches (55%), people’s recommendations (53%), self-learning (39%), and books/ magazine (16%). The most commonly used COVID-19 home remedies among this group were cloves (69%) and lemon (69%), followed by coconut (31%), eucalyptus oil (16%), traditional/ herbal cough syrup (7.5%), and turmeric (7.5%).

### PLS-SEM

**Fig. 1:**
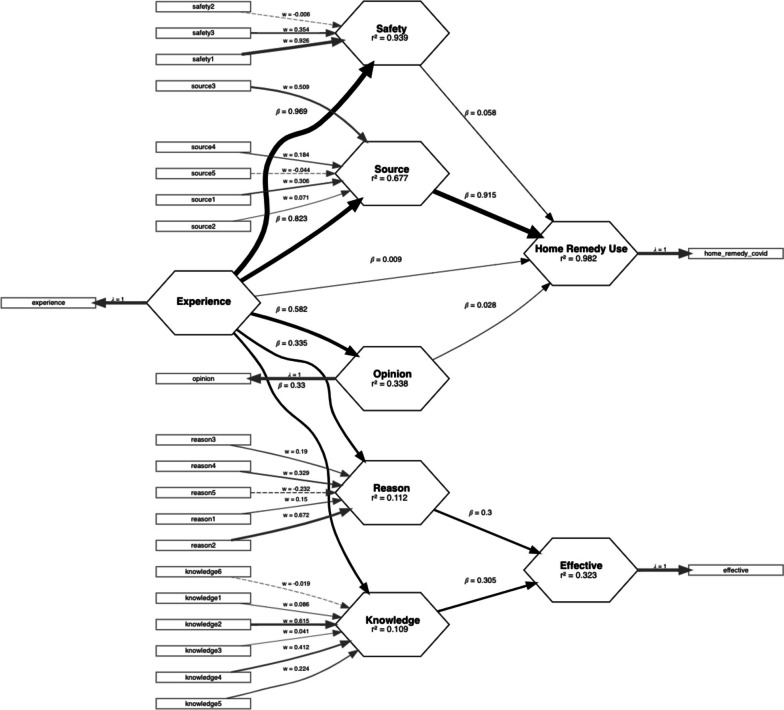
Partial least squares structural equation model of home remedy use of Brunei adult population

To further explore the results of the collected data, Partial Least Squares Structural Equation Modelling (PLS-SEM) was used to model the relationship between endogenous factors including ‘Experience’, ‘Safety’, ‘Source’, ‘Opinion’, ‘Reason’, and ‘Knowledge’ towards the two main outcomes – the intention of COVID-19 home remedy use and perceived effectiveness, among Brunei adult population which is shown in Fig. 1. The structural equation model demonstrated that ‘Experience’ appears to be a central endogenous variable that affects the decision on the use of COVID-19 home remedy. This relationship is mediated by ‘Safety’, ‘Source’, and ‘Opinion’ which have significant contributions to the outcome, accounting for 98.2% of the variance explained (R-square = 0.982). On the other hand, ‘Experience’ is also central to the relationship towards the perceived effectiveness of COVID-19 home remedy usage. We observed that this relationship is mediated by ‘Reason’ and ‘Knowledge’ which has an important contribution towards this outcome, accounting for 32.3% of the variance explained (R-square = 0.323). With the respective structural model, the path coefficients are presented in Table 10. After adjusting for confounding variables, we observed that ‘Source’ (*β* = 0.915) is the main enabler for home remedy use (Adjusted R-square = 0.982). While ‘Reason’ (*β* = 0.300) and ‘Knowledge’ (*β* = 0.305) are important contributors towards perceived effectiveness (Adjusted R-square = 0.321).

**Table 10:** Path Coefficients of PLS-SEM

	**Home Remedy Use**	**Effective**	**Reason**	**Opinion**	**Safety**	**Source**	**Knowledge**
**R**^**2**^	0.982	0.323	0.112	0.338	0.939	0.677	0.109
**Adj R**^**2**^	0.982	0.321	0.111	0.338	0.939	0.676	0.108
**Experience**	0.009	-	0.335	0.582	0.969	0.823	0.330
**Opinion**	0.028	-	-	-	-	-	-
**Safety**	0.058	-	-	-	-	-	-
**Source**	0.915	-	-	-	-	-	-
**Reason**	-	0.300	-	-	-	-	-
**Knowledge**	-	0.305	-	-	-	-	-

Abbreviation: *Home Remedy Use* Home remedy use, *Effective* Perceived effectiveness, *Experience* Previous experience of general home remedy usage, *Source* Home remedy ingredient source, *Safety* Perceived safety of home remedy use, *Opinion* Opinion of the effectiveness of home remedy use as an alternative or integrative approach, *Reason* Reason for using COVID-19 home remedy, *Knowledge* The knowledge source for COVID-19 home remedy

*R*^*2*^ R-square, *Adj R*^*2*^ Adjusted R-Square

Table 11 presents the measurement model whereby the reliability and convergent validity of the PLS-SEM model. Cronbach’s alpha statistics indicated that all the constructs had good to excellent internal consistency reliability except for the ‘Safety’ domain (alpha = 0.116). In the same vein, ‘Safety’ also had Average Variance Explained (AVE) lower than 0.5, indicating that the latent variance may not be completely captured by the items. However, these items were not dropped as they are conceptually important.

**Table 11:** Reliability of PLS-SEM

	**alpha**	**rhoC**	**AVE**	**rhoA**
**Experience**	1.000	1.000	1.000	1.000
**Opinion**	1.000	1.000	1.000	1.000
**Safety**	0.116	0.476	0.340	1.000
**Source**	0.965	0.966	0.851	1.000
**Reason**	0.803	0.790	0.452	1.000
**Knowledge**	0.776	0.811	0.428	1.000
**Home Remedy Use**	1.000	1.000	1.000	1.000
**Effective**	1.000	1.000	1.000	1.000

*Range* Alpha, *rhoC*, and rhoA should exceed 0.7 while AVE should exceed 0.5

*Abbreviation*: *Home Remedy Use* Home remedy use, *Effective* Perceived effectiveness, *Experience* Previous experience of general home remedy usage, *Source* Home remedy ingredient source, *Safety* Perceived safety of home remedy use, *Opinion* Opinion of the effectiveness of home remedy use as an alternative or integrative approach, *Reason* Reason for using COVID-19 home remedy, *Knowledge* The knowledge source for COVID-19 home remedy

## Discussion

### Prevalence

The study revealed a higher prevalence COVID-19 home remedy usage (72.2%) in Brunei compared to earlier reports in other countries during the 2020 pandemic outbreak. Iran and The Netherland reported 69% [34] and 68% [31], respectively, for complementary and integrative medicine use. South African university students and academic staff reported a significantly lower with traditional medicine use at 30.4% [35]. In contrast, Brunei’s study demonstrated a higher prevalence among those with higher education (76%) than those with lower education levels (24%). Despite being the first study of its kind on COVID-19 home remedy use in Brunei, the 70% prevalence of prior general home remedy use exceeds a 2007 study’s finding (59%) in Brunei [10] indicating a rising trend in home remedy as well as complementary and integrative medicine utilisation in modern healthcare.

### Perception, attitude, and practice of COVID-19 home remedy

This study explores the perception, attitude, and practice of COVID-19 home remedy of adults in Brunei using PLS-SEM. The result from the PLS-SEM suggests a substantial impact of general home remedy consumption experience on participants’ decisions to consume home remedy for COVID-19, with mediating factors such as perceived safety, ingredient accessibility, and participant opinions on the home remedies influencing this relationship. The findings suggest that participants’ trust in the safety of the home remedy sems from their past experiences in safely using its ingredients.

In the contemporary era, where knowledge is easily accessible, individuals seek knowledge on COVID-19 home remedy through various channels, including social media and the Internet. While social media constituted a notable knowledge source for our participants (30%), this study showed that a higher proportion acquired knowledge from family members (36%). A previous study on general home remedies in Germany found that consumers predominantly obtained information from family members (80%) [33]. Conversely, in a study involving pharmacists noted that patients learned about COVID-19 home remedy primarily through social media and media (73.5%) and self-decision (73.1%), surpassing information obtained through healthcare prescriptions after seeking CAM (65.9%) [36].

Furthermore, another prominent motivation for our participants in using home remedy is familial recommendation (32%). This postulates a crucial influence of personal motivation and close social networks, as only 16% reported using home remedy based on the recommendations from other individuals. A study investigating factors influencing COVID-19 preventive behaviour found that individuals can be socially influenced when trusted individuals in their social circle adhere to a social norm [37]. A global systematic review highlighted that people primarily turn to CAM due to their expectations of its benefits, discontentment with conventional medicine, and the perceived safety of CAM [38]. In addition, Western population tend to be internally motivated, while Asian populations lean towards external motivation influenced by social networks. In contrast, affordability, accessibility, and tradition play pivotal roles influencing factors for the African population [38]. A Hong Kong study on predictors of the intention to use Traditional Chinese Medicine applying the theory of planned behaviour exhibited that attitude towards Traditional Chinese Medicine was strongly influenced by subjective norms, perceived behavioural control, and knowledge, indicating the impact of advice from others in encouraging individuals to seek Traditional Chinese Medicine [39].

Interestingly, while a substantial portion of our participants utilised the COVID-19 home remedy to alleviate symptoms, they predominantly perceived it as less or not effective. Despite this perception, they majority considered it safe for use and continued its application, even with uncertainty regarding its compatibility with modern medicine and perceptions of reduced efficacy. These findings suggest a dual motivation, influenced both internally and externally, guiding individuals in their choice to resort to home remedies. Further research could explore deeper into distinguishing the relative influence of family compared to friends and strangers, particularly within the Asian population.

### Types of COVID-19 home remedy

Participants in this study consumed COVID-19 home remedies, aligning with a systematic review by Soltani et al. in 2022, which highlighted six individual types [40]. In contrast, our study found users reporting consumption of cloves, lemon, honey, coconut, eucalyptus oil, *habbatussauda,* traditional/herbal cough syrup, and turmeric. The subsequent discussion explores the usage of these home remedies in comparison with findings from various studies worldwide.

#### Cloves

Reportedly, a prevalent COVID-19 home remedy in Brunei with 37% usage. Other studies reported varying prevalence such as 50.5% usage as herbal tea reported by Bhol et al. in 2021, 2.5% as a clove solution reported by AlNajrany et al. in 2021, and unspecified prevalence by Mshana et al. in 2021 [40]. In an in-silico investigation, clove components including black pepper, and ginger were found potentially suitable to combat COVID-19 [41]. Cloves, also known as, *Syzygium aromaticum L.,* is traditionally used in Asia in varying forms such as tea, aromatherapy, oil, or mixed with honey to treat upper-respiratory tract illnesses to ease coughs, colds, asthma, bronchitis, and sinusitis, as well as being chewed to treat sore throat, pharynx inflammation and severe coughing [42]. With previous usage for other respiratory ailments, cloves were supported scientifically with potentials to treat COVID-19.

#### Lemon

Lemon, with a 37% prevalence, parallels clove consumption in this study. Systematic review data, however, shows higher COVID-19 home remedy usage prevalence: 54% by Alotiby et al. in 2021, 50.5% by Bhol et al. in 2021 as part of herbal tea, and 45.2% by AlNajrany et al. in 2021 [40]. In silico research indicates *Citrus limon* ‘s potential antiviral activity against COVID-19 [43]. Lemon’s multifaceted properties were utilised in pharmaceutical, cosmetic and culinary applications, historically addressing scurvy and boosting vitamin C levels, as mentioned by Benedec et al. in 2017 whereby in Romanian traditional medicine a mixture of essential oil from *C. limon* and sugar would be used to beat cough [43].

#### Honey

Honey, the third most prevalent COVID-19 home remedy at23% aligns with systematic review data showing a higher percentage of consumption by the participants of Alotiby et al. (84%) in 2021, as part of a herbal tea in Bhol et al. (50.5%) in 2021, AlNajrany et al. (46.1%) in 2021, d’Arqom et al. (30%) in 2021, and an unspecified prevalence by Nguyen et al. in 2021 [40]. In Pakistan, a placebo-controlled randomised clinical trial explored honey and *Nigella sativa*’s therapeutic properties for COVID-19, including antiviral, anti-inflammatory, and immunomodulatory properties, revealing improved symptoms recovery, accelerated virus clearance, and reduced mortality rate among patients with COVID-19 compared to a placebo [44]. Honeybee and propolis boast medicinal properties, including potential antiviral effects against COVID-19 according to an in-silico study, however, more studies are needed to confirm their clinical efficacy [45]. In a systematic review of in-silico and clinical studies, the in-silico studies presented propolis of having potentially anti-viral effect on SARS-CoV-2, yet, limited clinical evidence are available to show that propolis and honey could potentially improve COVID-19 symptoms and faster viral clearance [46]. Similarly, another review suggested honey as an adjuvant in the treatment of COVID-19 infection, stating its antiviral properties related to its antioxidant and anti-inflammatory attributes and urging more clinical trials in the future [47]. Supplemental vitamins C, vitamin D, and natural honey, according to a review, seem promising to alleviate symptoms and disease progression alongside conventional treatment of COVID-19, however, more extensive clinical validation is essential [48].

#### Coconut

This study reported the 4.6% prevalence of coconut consumed as home remedy. Coconut (*Cocos nucifera L.*) is known to have isotonic properties, could treat many infections, has many health benefits, possesses antiviral properties, and can also reinforce the immune system [49]. A randomized controlled trial in the Philippines revealed virgin coconut oil when added to the meals of probable suspected COVID-19 cases might be effective in enhancing COVID-19 recovery [50]. Another study also supported the use of coconut oil as a potential complementary therapy for COVID-19 for its antiviral properties and proven food safety [51].

#### Eucalyptus oil

The prevalence of eucalyptus oil utilisation for COVID-19 in Brunei was 3.7%, while a systematic review notes a slightly higher prevalence as reported by d’Arqom et al. in 2021 (5%) and an unspecified prevalence reported by Mshana et al. in 2021 [40]. A widely studied essential oil, eucalyptus oils were commonly used in aromatherapy and inhalation. It typically contains eucalyptol, that therapeutic properties such as antitussive, mucolytic, anti-inflammatory, and antimicrobial, and menthol, which also has an antitussive effect and helps to ease breathing by reducing dyspnoea sensation [18]. Despite the remedial effect, essential oils lacks sufficient clinical evidence for curative purposes in COVID-19 treatment, offering only symptomatic relief without reduce the virus contagiousness [18].

#### Habbatussauda

This study reported a 3.5% use of *habbatussauda* (black cumin or black seed), scientifically termed *Nigella sativa*, with traditional and religious importance in Chinese, Indian, Arab, and Islamic Medicine, as well as biblical references as a spice and Islamic *Hadith references* as a prophetic medicine [15]. Iran reported a higher prevalence (74.3%) of its consumption [34]. Varying prevalence were reported in a systematic review of COVID-19 home remedy, with Alotiby et al. reporting the highest prevalence (63%) followed by AlNajrany et al. (26%), Teke et al. (5.6%) [40]. Supported by pharmacological studies, *Nigella sativa* demonstrates diverse properties, including antitussive, antioxidant, hepatoprotective, neuroprotective, gastroprotective, immunomodulator, analgesic, antimicrobial, anti-inflammatory, spasmolytic, and bronchodilator activities [12, 15]. A native to North Africa, Eastern Mediterranean, the Indian subcontinent, and Southwest Asia, its traditional use in Africa encompasses treating asthma, cough, bronchitis, rheumatoid arthritis, diabetes, and hypertension, and enhancing the immune system to fight illnesses, suggesting potential anti-coronaviral properties [12]. Given COVID-19;s viral nature and lung inflammation manifestation, *Nigella sativa’s* antimicrobial features may contribute to treatment, potentially reducing severity and expediting recovery.

#### Turmeric

This study, notes a 2.2% prevalence of turmeric consumption, contrasting with higher rates in a systematic review: reported by Bhol et al. in 2021 it was made as turmeric water or milk (67.7%) and as part of herbal tea (50.5%), by Alotiby et al. in 2021 (31%), 9.9% prevalence reported by Teke et al. in 2021, and unspecified prevalence by Alyami et al. in 2020 [40]. *Curcuma longa*, indigenous plant in South Asia and grown commonly in East and West Africa, were used for cooking and medicine, and held pharmacological values such as anti-inflammatory, anti-ulcer, antioxidant, anti-diabetic, anticoagulant, antifertility, anti-neoplastic, antimicrobial, antiviral, wound healing, cardiovascular protective, hepatoprotective and immunostimulant properties, whereby it was previously being studied for prophylaxis and management of SARS Coronavirus and is currently being studied for SARS-CoV-2 [12]. A clinical trial showed early recovery from COVID-19 symptoms, reduced hospitalisation, and fewer deaths in moderate to severe COVID-19 patients given curcumin and piperine (found in turmeric and black pepper, respectively) [52].

These home remedy ingredients can be commonly found in a household kitchen, considering that the ingredients can also be used for culinary purposes. Most of these ingredients possess therapeutic properties, including antiviral and anti-inflammatory properties. These properties are crucial in the treatment or symptom management of COVID-19 symptoms as it manifests as a respiratory illness and may involve the inflammation of the lungs. As SARS-CoV-2 is a novel virus, most of the home remedies suggested aimed to improve the overall well-being of patients with COVID-19 and to accelerate recovery, as well as to reduce the long-term impact of the disease.

### Self-management guide with COVID-19 home remedy

Learning from Italy, the efficient management of mild-to-moderate COVID-19 patients at home, it reduces the pressure on their healthcare system minimising the socio-psychological impact on patients, albeit the lack of experiments to support the observed management with home therapy at that time [53]. Revised in 2021, Brunei Darussalam’s Ministry of Health published an online guide of the COVID-19 self-management at home for home-isolation patient, which included simple symptom management with over-the-counter medications and simple home remedies. For instance, paracetamol for fever, cough syrup for cough, lozenges or gargling with salt water for sore throat, and antihistamine or nasal decongestant for rhinitis [54]. While the guidebook incorporated various at-home self-management suggestions, it still lacked comprehensive recommendations on home remedies. Therefore, the findings from this study could contribute to fill the knowledge gap in the guidebook as the commonly used COVID-19 home remedies were identified in the Brunei adult population, supported by empirical evidence.

### Limitations

The population of the study may only be true to patients with the same socio-characteristics. Since Brunei has a diverse cultural background mix, the cultural impact may only be generalised to the Bruneian population and around the ASEAN regions. There was not enough data needed to analyse how the remedies were being consumed. The study was also limited to only people who have contracted COVID-19, and this study did not investigate those who use home remedy to prevent COVID-19 infection and to recover after infection, such as long-COVID or post-COVID conditions. Furthermore, this study did not examine the co-morbidities such as hypertension and diabetes mellitus among the participants of study. It would be recommended in further research that these limitations be addressed to provide deeper knowledge on the topic of COVID-19 home remedies.

## Conclusion

The outcome of this study establishes the baseline prevalence of COVID-19 home remedy consumption and its perceived effectiveness among Bruneian residents, as well as comparing the previous study on Brunei’s general use of traditional medicine. An approach using the PLS-SEM presenting the perception, attitudes, and practices of the participants might be beneficial for future research to predict the health-seeking behaviour of Bruneian adult population. PLS-SEM determined that participants’ past experience with general home remedies significantly influences their decision to use home remedies for COVID-19, mediated by perceived safety, ingredient accessibility, and opinions, suggesting trust is the safety based on prior ingredient use. The study also identified the types of COVID-19 home remedy consumed by the study population, which are comparable to other studies and supported by experimental and clinical evidence. The present findings could help nurses as well as other healthcare professionals in Brunei understand the practise of Bruneian adults of consuming home remedies for COVID-19. Future reviews and international standardised terms of use in home remedy research might be beneficial to tackle the challenges in clearly defining the literature on home remedy, traditional medicine, herbal medicine, and other related CAM. Potential directions of related research might be able to categorise the components of COVID-19 home remedy that could be used as a preventive, prophylaxis, curative and rehabilitative home remedy. As the world continues to live with the impact of COVID-19, more research questions and opportunities can be explored to contribute to the knowledge of COVID-19 and home remedy.

## Data Availability

The datasets used and/or analysed during the current study are available from the corresponding author on reasonable request.

## Abbreviations

COVID-19: Coronavirus Disease 2019
PLS-SEM: Partial Least Squares Structural Equation Modelling
CAM: Complementary and alternative medicine
